# From Phytotoxin to Cell-Death Probe: Ophiobolin A and Related Sesterterpenoids in Membrane Stress and Non-Apoptotic Cell Death

**DOI:** 10.3390/molecules31071206

**Published:** 2026-04-05

**Authors:** David Aebisher, Izabella Wilk, Dorota Bartusik-Aebisher

**Affiliations:** 1 Department of Photomedicine and Physical Chemistry, Medical College, University of Rzeszów, 35-025 Rzeszów, Poland; 2 English Division Science Club, Medical College, University of Rzeszów, 35-025 Rzeszów, Poland; iw135750@stud.ur.edu.pl; 3 Department of Biochemistry and General Chemistry, Medical College, University of Rzeszów, 35-025 Rzeszów, Poland

**Keywords:** Ophiobolin A, fungal sesterterpenoids, electrophilic natural products, structure–activity relationships, covalent lipid modification, paraptosis, apoptosis-resistant cancer, calmodulin inhibition, anticancer natural products, ferroptosis

## Abstract

Ophiobolin A is a fungal sesterterpenoid initially characterised as a phytotoxin but progressively investigated for its biomedical significance due to its potent and mechanistically characteristic cellular activities. In this review, Ophiobolin A is discussed within the wider landscape of natural products as a source of bioactive molecular scaffolds, and current knowledge on its structural features, biosynthesis, chemical synthesis, semi-synthetic modification, and in vitro biological applications is summarised. Evidence drawn from chemical, biochemical, and cell biology studies is integrated to describe the distinctive 5-8-5 tricyclic scaffold, the electrophilic dicarbonyl motif, and their roles in covalent modification of cellular components. Collectively, the reviewed evidence underscores that Ophiobolin A and its derivatives trigger both apoptotic and non-apoptotic cell death pathways. These include paraptosis-like death, which is a regulated form of cell death not associated with apoptosis that is defined by major cytoplasmic vacoulisation. This commonly occurs in apoptosis-resistant cancer models via disruption of membrane lipid homeostasis, calmodulin-dependent signalling, mitochondrial function, and proteostasis. Structure–activity relationship studies show that modulation of electrophilicity, oxidation state, and peripheral functionality enables tuning of potency, selectivity, and traceability while retaining key phenotypes. In addition to anticancer effects, antimicrobial and anti-inflammatory activities are also briefly summarised. Taken together, the literature supports Ophiobolin A as a useful molecular probe for considering cell death mechanisms and as a chemically complex yet suitable starting point for derivative development, while reinforcing the need for improved selectivity, delivery strategies, and in vivo validation to further translational potential.

## 1. Introduction

Both as approved drugs and as chemical frameworks that provide a structural basis for semi-synthetic or fully synthetic analogues, an extremely large share of clinically useful chemical matter continues to be provided by natural products. The ability of evolutionarily selected metabolites to involve complex biology with high ligand efficiency and structural uniqueness is reflected by large-scale analyses of modern approvals highlighting “natural product” origin as a dominant theme in oncology and anti-infectives [1]. Simultaneously, the discovery process has been strengthened by methodological advances in genome mining, metabolomics, and high-content phenotypic screening, which has enabled faster linkage of bioactivity to structure and biosynthetic origin [2]. Fungal secondary metabolites occupy an advantaged position in this field, as chemically dense and stereochemically rich frameworks are regularly produced by fungi. They often contain electrophilic functionalities and unique ring systems, which are highly effective at disrupting eukaryotic signalling and membrane homeostasis.

Within the class of fungal terpenoids, ophiobolins represent a family of C25 characteristic sesterterpenoids. They were initially recognised for their phytotoxicity but are increasingly investigated for their biomedical potential. Marine-derived fungi are a widely recognised source of chemically reactive anticancer frameworks, especially terpenoids that elicit non-apoptotic stress responses. Ophiobolins are consistent with this broader marine-fungal pattern of cytotoxicity, which is led by membrane and organelle disruption instead of single-protein inhibition [3]. The first structural characterisation of the class can be traced to classic isolation and structure elucidation work by Nozoe et al., who established a new framework in terpene chemistry when they reported the novel carbon skeleton of ophiobolin in the mid-1960s [4]. The family of plant-pathogenic and endophytic fungi, such as *Bipolaris* and *Drechslera*, has been expanded in subsequent studies. Additionally, the wide range of activities in plants, microbes, and mammalian systems has been emphasised in comprehensive biological summaries. This positions Ophiobolin A (OpA) as a key reference for mechanism-of-action studies [5]. Many natural congeners are now catalogued in modern overviews, which highlight how bioactivity profiles can be significantly reshaped by stereochemical and oxidation-state differences [6,7]. Even within the ophiobolin family, OpA is chemically unique. OpA is characterised by a distinctive tricyclic 5-8-5 fused ring system. It consists of a cyclooctane embedded within a compact polycyclic structure, coupled with a dense array of oxygenated functionality and reactive carbonyl motifs. These features collectively establish OpA as a conformationally constrained, three-dimensional molecular scaffold [6]. Both the structural conversion of the 5-8-5 core and the variation in peripheral oxidation patterns across producing strains can be rationalised by biosynthetic analyses. This provides clarification for this architecture arising from specialised sesterterpene cyclisation pathways, frequently involving bifunctional enzymes that pair prenyl chain assembly and cyclisation [8].

OpA’s integration of a rigid polycyclic backbone and selectively positioned electrophiles is especially noteworthy from a medicinal chemistry perspective. OpA can support both high-affinity noncovalent recognition and covalent modification of nucleophilic protein residues. These are attributes that can translate into pronounced, pathway-selective phenotypes but also require cautious profiling for off-target reactivity. Previously, OpA’s inhibition of calmodulin-dependent processes was seen to drive mechanistic interest. It was demonstrated in early biochemical studies that calmodulin-activated cyclic nucleotide phosphodiesterase is strongly inhibited by OpA [9], whereas in follow-up work, calmodulin inhibition was linked to phytotoxicity, and specific lysine residues involved in binding and inactivation were mapped [10,11]. In modern target-discovery efforts, this ability for covalent or quasi-covalent engagement has been persistently observed, including in recent chemoproteomic studies that facilitate a broader interrogation of proteome-wide reactivity and downstream effects on cancer cell viability [12]. Cell biology studies have simultaneously demonstrated that in apoptosis-resistant contexts, OpA can stimulate non-apoptotic cell death programmes. For example, paraptosis-like death, a non-apoptotic cell death phenotype associated with vacuolisation, has been observed in glioblastoma models. This positions OpA as a promising lead for the treatment of challenging malignancies [13]. These developments are synthesised in current reviews, and the evolving therapeutic framework is highlighted, which is that, through mechanism-guided optimisation and derivative design, a fungal phytotoxin scaffold is being recontextualised. It is now being considered for therapeutic application as a starting point for anticancer and other potential medical applications [14].

## 2. Materials and Methods

**Figure 1:**
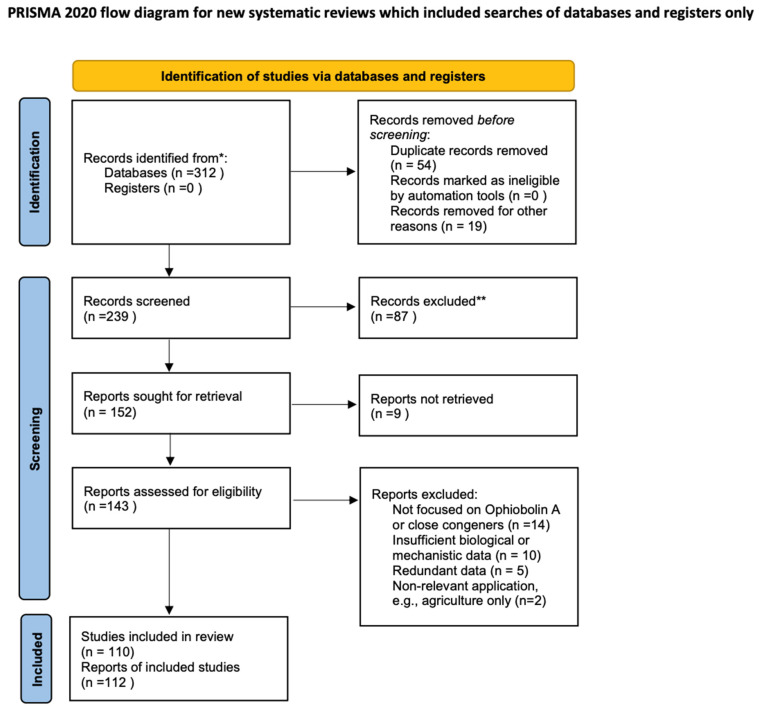
PRISMA flow diagram of literature selection. Flowchart showing the identification, screening, eligibility assessment, and inclusion of studies retrieved from PubMed and PMC for this review. The mark (*) means the total number of recognized records. The mark (**) means the total number of excluded records.

PRISMA guidelines for literature identification and selection were followed in this review. Studies on Ophiobolin A and related congeners using structure-, mechanism-, and application-focused keywords were searched for on PubMed and PubMed Central (PMC). Articles were screened by title and abstract, followed by evaluation of the full text for relevance to biosynthesis, chemical modification and biological activity in vitro. The PRISMA flow diagram in Figure 1 summarises the study selection process.

## 3. Biosynthetic Origin, Structural Features, and Ecological Roles of Ophiobolins

Ophiobolins are sesterterpenoids most frequently derived from plant-associated filamentous fungi. Mangrove-derived endophytic fungi represent an important source, frequently producing several new ophiobolin congeners from a single strain, emphasising their value for scaffold diversification [15]. Ophiobolins act as phytotoxins that cause necrotic lesions and contribute to disease symptoms in cereals and grasses in classic producer genera, including *Bipolaris* spp. and *Drechslera* spp., such as *B. maydis*, *B. sorokiniana*, and *B. setariae* [16]. 3-anhydro-ophiobolin A and 6-epi-ophiobolin A were put forward as significant determinants of pathogenicity in *Bipolaris setariae*, supporting a direct ecological role in host colonisation [17]. Genetics have identified many biosynthetic gene clusters and a bifunctional ophiobolin synthase that assists ophiobolin biosynthesis and diversification within *Aspergillus* spp. This establishes *Aspergillus* spp. as another important source, which includes mangrove-sourced *Aspergillus ustus* and endophytic *Aspergillus calidoustus* [18]. Ophiobolin K from *A. ustus* displays antibacterial and antifungal activity, suggestive of an ecological defence function. This demonstrates that ecologically, beyond plant pathogenesis, ophiobolins can function as chemical defence agents in microbial competition [19]. These ecological roles explain why ophiobolins are highly active, membrane-reactive cytotoxins in vitro, and why viable synthetic pathways to the supply of OpA and its related congeners for pharmacology include fermentation, strain engineering, and semi-synthesis from fungal extracts [20].

OpA is a C25 sesterterpenoid underpinned by a distinctive 5-8-5 tricyclic carbon skeleton (A-B-C ring system) that rigidly displays substituents and supports semi-synthetic modification for therapeutic lead discovery [21]. OpA’s electrophilic unsaturated 1,4-ketoaldehyde/1,4-dicarbonyl region, which consists of an enal adjacent to a ketone, is the main bioactivity hotspot. Pyrrole-type covalent adducts are formed when this motif reacts with primary amines, and covalent modification of phosphatidylethanolamine (PE) links it to cytotoxicity [22]. It is demonstrated in recent semi-synthetic structure–activity relationship (SAR) studies that both potency and selectivity correlate with preservation of the electrophilic unsaturated dicarbonyl motif [23]. Relative to other ophiobolins, oxidation patterns, double-bond placement, and stereochemistry, which regulate electrophilicity and 3D shape, differ between congeners, although they share the same 5-8-5 core. These variations are related to distinct anti-proliferative, phytotoxic, and antimicrobial profiles in reviews contrasting OpA with ophiobolins B/C and other members [6]. Stereodefined access for probe design is provided by total syntheses, and bicyclic derivatives that retain anticancer activity, providing a foundation for scaffold-based medicinal chemistry, are yielded by pharmacophore-guided simplification [24]. OpA efficiency against drug-resistant cells in glioblastoma models has prompted derivative programmes. Recent OpA derivatives with improved activity under tumour-microenvironment conditions show that adjusting peripheral oxidation while retaining the reactive dicarbonyl can improve performance without disrupting the ophiobolin topology [25]. This enables synthetic–biological iteration for in vitro translation.

**Figure 2:**
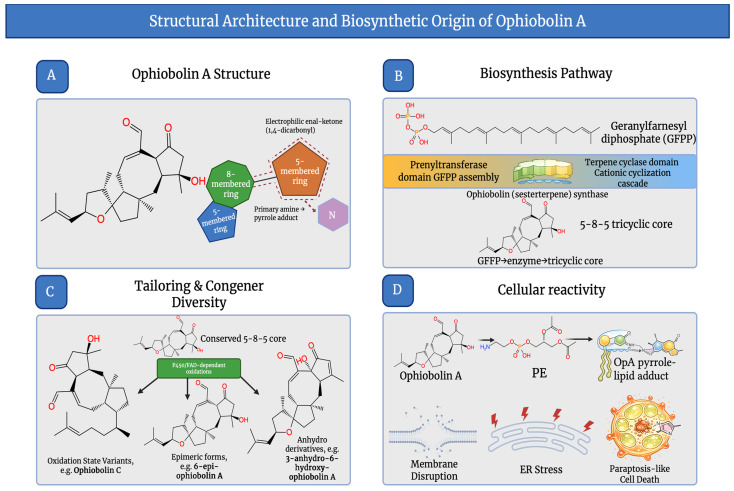
Structural architecture and biosynthetic origin of Ophiobolin A. (**A**) Core chemical structure highlighting the 5–8–5 tricyclic scaffold and electrophilic motif; (**B**) Biosynthetic formation of the tricyclic core from geranylfarnesyl diphosphate (GFPP); (**C**) Tailoring reactions generating ophiobolin congeners; (**D**) Cellular reactivity leading to membrane disruption, ER stress, and paraptosis-like cell death. Created in BioRender. Wilk, I. (2026) https://BioRender.com/49l1lck.

OpA is biosynthesised from geranylfarnesyl diphosphate (GFPP) and belongs to the fungal sesterterpenes (C25). Terpene cyclisation catalysed by a bifunctional sesterterpene synthase, commonly referred to as ophiobolin synthase, constructs the characteristic 5-8-5 tricyclic. GFPP is assembled by a prenyltransferase domain. A cationic cyclisation and rearrangement cascade that forms the 5-8-5 core is triggered by a terpene cyclase domain [8], as shown in Figure 2. In *Aspergillus* and *Bipolaris*, biosynthetic gene clusters (BGCs) that encode this machinery have been characterised. In several *Bipolaris* spp., comparative genomics has localised conserved ophiobolin BGC regions, supporting an evolutionarily conserved biosynthetic strategy to produce these bioactive metabolites [26]. Studies demonstrate that in *Aspergillus ustus* production of ophiobolin relies on the C25 synthase gene, together with coordinated activity from additional terpene-related clusters. This emphasises pathway-level regulation instead of single-enzyme limitation [18]. Current genome-guided surveys of *Aspergillus* secondary metabolism suggest that terpenoid biosynthetic diversity continues to grow, strengthening the usefulness of cluster-driven exploration for new ophiobolin congeners [27]. Subsequently, oxidation patterns that correlate with OpA and derivative bioactivity in vitro could be introduced by tailoring enzymes, particularly cytochrome p450 monooxygenases and flavin-dependent oxidases [28]. Light conditions can regulate OpA production in *Bipolaris maydis*, which is relevant to fermentation optimisation for supplying material for semi-synthesis and pharmacology, demonstrating regulation is environmentally responsive [29]. These structure–biosynthesis–regulation relationships are summarised in Table 1. Furthermore, scalable access for medical and mechanistic studies is supported by synthetic biology efforts that establish de novo biosynthesis of ophiobolin-type sesterterpenoids [21].

## 4. Chemical Synthesis and Semi-Synthetic Derivatives of Ophiobolin A

### 4.1. Total Synthesis Strategies and Stereochemical Challenges

The total synthesis of OpA is challenging due to the regulation of dense stereochemistry. In many structural analogues, a spirocyclic ether (5-8-5-5) is incorporated into the framework, while the 5-8-5 fused carbocyclic core is assembled. Due to unfavourable entropic contributions, transannular interactions, and alternative rearrangement pathways during medium-ring formation, the highly substituted eight-membered ring is a constant synthetic limitation [30]. Early ophiobolin nucleus construction studies that established feasible strategies for building the tricyclic framework are considered key milestones [32]. A major recent advance was the convergent and enantiocontrolled total synthesis of (+)-OpA by Tsuna et al. It combined stereoselective Hosomi–Sakurai cyclisation to establish the spiro-fused C-D ring system, fragment coupling to install the A ring, and olefin-metathesis-mediated ring formation. This demonstrated a framework for constructing the full ophiobolin structure [24,30]. In recent years a simplified platform that resolved the spirocyclic tetrahydrofuran and stereochemical problems with reduced step count was provided by a 14-step total synthesis of (+)-6-epi-ophioblin A [31]. With respect to yield and scalability, total synthesis continues to be multi-step with modest overall yield. Therefore, fermentation or isolation followed by semi-synthesis is frequently preferred for providing OpA and its analogues for in vitro pharmacology. Nevertheless, synthetic throughput can be improved by more recent cascade or cyclisation strategies that quickly access 5-8-5 tricycles, facilitating medicinal chemistry diversification under conditions of inadequate natural supply [33].

### 4.2. Semi-Synthetic Modification Axes for Biological and Medicinal Studies

Semi-synthetic approaches are essential to translating OpA into practical in vitro tools and medicinal leads. The natural product is generally poorly water-soluble as well as highly electrophilic. However, its activity can be strongly impacted by small structural changes. OpA’s covalent reactivity, exemplified by the pyrrolylation of PE, and its cytotoxicity are predominantly determined by a first modification axis, which targets functional groups around the defining unsaturated aldehyde/ketone, 1,4-dicarbonyl, region [7]. The ketone and enal/aldehyde moieties have been systematically modified in recent semi-synthetic SAR studies. This involved selective reduction, oxidation, and derivatisation, showing that reducing electrophilicity can improve handling. However, this often decreases biological activity, which is valuable for separating reactivity from activity when designing probes [23]. Solubility and chemical stability aim to be improved by a second axis, without fully eliminating the pharmacophore. Common strategies include introducing ester/ether handles, installing polar linkers, or formulating prodrug-like derivatives, such as amino-acid or ionisable motifs, to increase aqueous solubility and decrease nonspecific compound loss during administration. These strategies are commonly used for poorly soluble electrophiles and can be optimised for derivatisation campaigns [34]. Adjusting of polarity and reactivity while preserving anti-proliferative activity against apoptosis-resistant cancer cells can be enabled by converting the reactive motif into unsaturated ester derivatives and even dimeric constructs in OpA-specific work [35]. Lastly, analogue-library generation directly from isolated OpA is supported by semi-synthesis. Derivatives with enhanced activity under tumour-microenvironment conditions have been produced from focused libraries that vary oxidation state, electrophile presentation, and linker design, generating traceable sets for mechanism-of-action and lead optimisation studies in vitro [25].

### 4.3. Structure–Activity Relationships Governing Electrophilicity and Cytotoxicity

**Figure 3:**
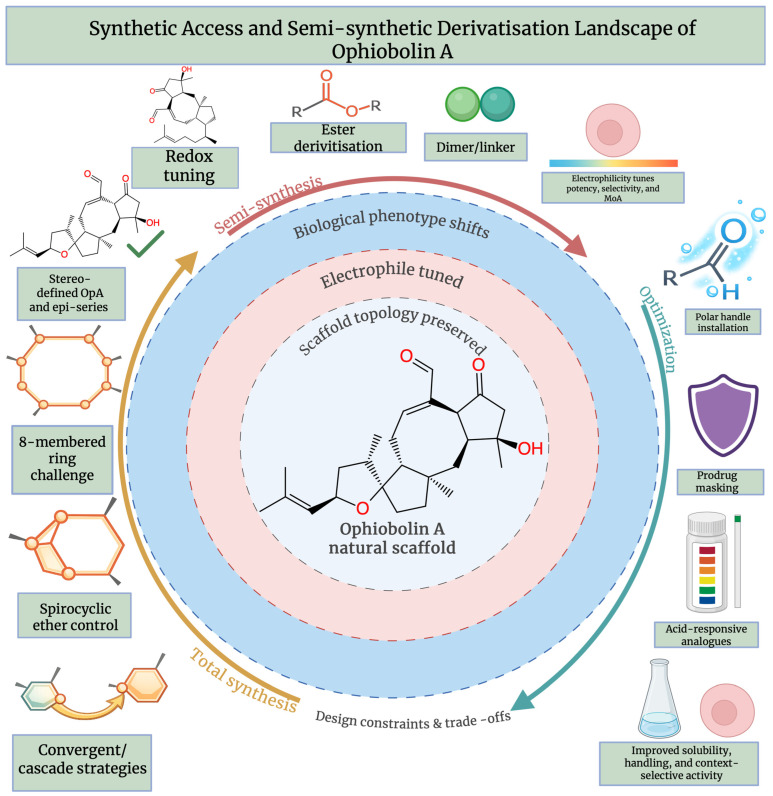
Synthetic access and semi-synthetic derivatisation landscape of Ophiobolin A. The preserved 5–8–5 Ophiobolin A scaffold acts as a hub for total synthesis and semi-synthetic strategies that adjust electrophilicity, redox state, and polarity, leading to biological phenotype shifts and optimisation of potency, selectivity, and usability. Created in BioRender. Wilk, I. (2026) https://BioRender.com/adtasa2.

Cytotoxicity is constantly traced to preservation of an electrophilic C5, C21 “dicarbonyl” region that functionally links the A-ring enone/ketone chemistry with the C21 aldehyde in SAR studies of OpA. When comparing natural congeners and early semi-synthetic analogues, studies indicate that alterations in stereochemistry or conjugation around this motif, e.g., 6-epi- and anhydro-OpA, significantly decrease activity. However, removing one carbonyl, such as by the reduction of the C21 aldehyde, can abolish activity completely. This suggests that this electrophile set is an essential requirement for marked anticancer effects. At the mechanistic level the aldehyde is not just a functionalisation site, but together with the adjacent carbonyls it also allows covalent chemistry in cells. With primary amines, OpA can undergo Paal–Knorr-type pyrrolyation, and cytotoxic covalent adducts can be formed with PE, which is a link correlated with paraptosis-like death in apoptosis-resistant cancer models [36]. Covalent modification of nucleophilic residues, such as lysines via Schiff-base-type chemistry, is also described in target-directed studies [12]. Modern derivatisation that selectively varies oxidation state at the ketone and the unsaturated aldehyde further supports the conclusion that adjusting electrophilicity tunes cytotoxicity toward breast cancer and glioblastoma stem-like cells [23]. Thus, pharmacophore proposals highlight the 1,4-keto or unsaturated aldehyde electrophile set with greater tolerance for modifications at the distal side chain in addition to the correct C5/C6 stereochemistry and topology of the A/B ring system and supportive C3 oxygenation. More generally, sesterterpenoids are increasingly acknowledged as a pharmacologically relevant class in oncology, supporting SAR efforts aimed at maintaining anticancer activity whilst reducing nonspecific reactivity [37]. OpA-focused SAR analyses do not identify an epoxide as a critical feature. Epoxides are best regarded as conformation or reactivity modulators when introduced in derivatives, rather than primary cytotoxic drivers [38]. The key electrophile-based modification strategies, design rationales, and associated biological consequences described above are summarised in Table 2 and Figure 3.

## 5. Molecular Targets and Mechanism of Action of Ophiobolin A

### 5.1. Membrane and Cytoskeletal Disruption via Covalent Lipid and Protein Engagement

OpA and semi-synthetic derivatives are commonly positioned as covalent, mechanism-guided leads in clinically orientated in vitro studies. Strong anticancer phenotypes and toxicity risks can be explained by their electrophilic motifs, which can disrupt membranes and cytoskeletal signalling. Disturbance of early secretory pathway function is known to increase endoplasmic reticulum (ER) stress and proteostasis collapse, offering a complementary framework for understanding ophiobolin-induced cytotoxicity [42]. Lipid targeting is a key membrane mechanism. OpA generates a pyrrole-containing covalent adduct with PE in human cells. This directly modifies a predominant inner-leaflet phospholipid and most likely alters bilayer structure, curvature, and the function of adjacent membrane proteins [22]. With respect to the cytoskeleton, calmodulin (CaM) is directly inhibited by OpA. CaM lysines that covalently bind OpA, specifically Lys-75 and Lys-148, were identified by site-directed mutagenesis and showed the primary inhibitory site to be Lys-75. Calcium ion (Ca^2+^)-dependent, effectively irreversible CaM inactivation was further supported by kinetic work [11]. This is significant because CaM is a vital regulator of actin dynamics. CaM has two ways of controlling actin organisation. The first is through the regulation of phosphatidylinositol-4,5-bisphosphate synthesis. The second is through the signalling of Ca^2+^/CaM-dependent myosin light chain kinase that modulates contractility and adhesion dynamics [43,44]. In line with these mechanistic connections, rapid, significant remodelling of F-actin, with reduced proliferation and migration, was exhibited in glioblastoma models treated with OpA in addition to a paraptosis-like vacuolisation process [13]. Collectively, membrane microenvironments can be destabilised by PE adduction, whilst actin assembly and force generation can be disrupted by CaM blockade. The combined effects can decouple the plasma membrane from the actin cortex, disrupt intracellular trafficking, and reduce cell motility. A reasoned route to derivatives with improved selectivity for therapeutic applications in synthesis efforts is tuning electrophilicity or sterics to favour PE versus CaM reactivity. Given that certain tumours externalise PE, OpA-like molecular frameworks have been described as chemical probes or payloads. This is because OpA can make use of this membrane lipid profile, although off-target concerns are also raised by covalent lipid chemistry [45].

### 5.2. Engagement of Multiple Regulated Cell Death Pathways

**Figure 4:**
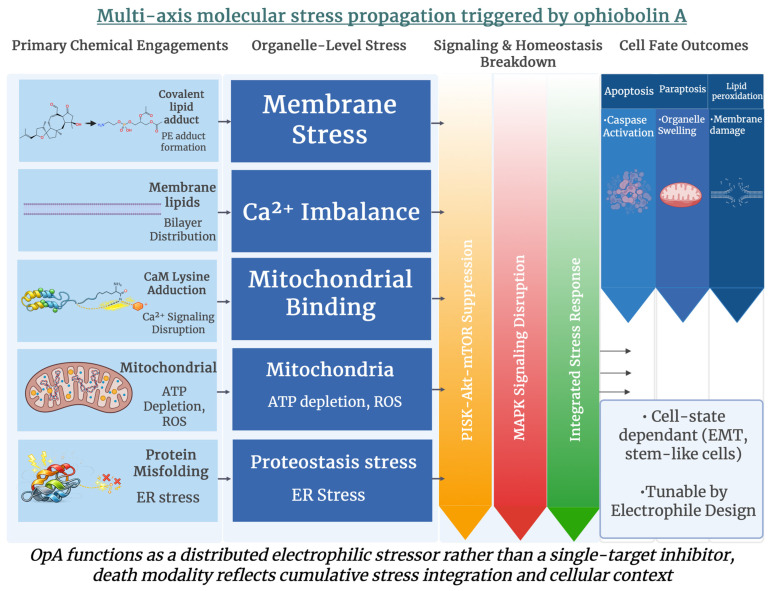
Multi-axis molecular stress propagation triggered by Ophiobolin A. Ophiobolin A induces parallel membrane, Ca^2+^, mitochondrial, and proteostasis stresses that converge on signalling collapse and integrated stress responses, leading to apoptosis, paraptosis, or lipid-peroxidation-associated cell death in a context-dependent manner. Created in BioRender. Wilk, I. (2026) https://BioRender.com/06wc560.

OpA and semi-synthetic derivatives can push cancer cells into distinct cell death pathways. This is useful when a tumour has inactivated one pathway and makes them appealing in medicinally orientated in vitro work. OpA can activate intrinsic (mitochondrial) apoptosis in apoptosis-proficient cells, whereas OpA induces mitochondrial compromise with autophagy: integration of apoptotic signalling with downstream caspase activation in melanoma [46]. Many ophiobolins, such as A, B, C and K, trigger apoptotic death at nanomolar concentrations in leukaemia-focused screens, confirming that apoptosis is a readily targetable outcome for this scaffold class [47]. Ophiobolin O alternatively exemplifies how structurally related congeners can direct cell death. This occurs through mitogen-activated protein kinase (MAPK)-linked apoptosis and by the inhibition of the cell cycle in breast cancer cells, including in chemo-resistant contexts [48,49,50]. As many solid tumours resist caspase-dependent death, non-apoptotic pathways are equally important, as shown in Figure 4. An apoptosis-bypass option is offered by paraptosis, which is defined as a programmed, caspase-independent vacuolating death with swelling and dilation of mitochondria and sometimes the endoplasmic reticulum. Paraptosis is progressively recognised as a therapeutically significant alternative to apoptosis, especially in apoptosis-resistant tumours, and is commonly stimulated by natural products that target organelle homeostasis [51]. MAPKs regulate paraptosis, and apoptosis-inducing protein-1 (AIP-1/Alix) inhibits it [52]. These phenotypes are connected by organellar stress biology. Continued unfolded-protein response signalling can shift from adaptation to pro-death outputs [53]. Meanwhile, reactive oxygen species (ROS)-driven lipid peroxidation and bioenergetic failure are amplified by mitochondrial redox imbalance [54]. In this context reactive oxygen species (ROS) accumulation and adenosine triphosphate (ATP) depletion would be expected to be intensified by diminishing of mitochondrial respiratory capacity with disruption of complex IV-dependent electron transport. This reinforces potentiating bioenergetic stress as opposed to acting as an isolated cytotoxic trigger [12]. The observed integration of redox imbalance, organellar dysfunction, and controlled cell death outcomes described for OpA-treated cells aligns with such a mitochondrial respiratory interference. A complementary, iron-dependent lipid-peroxidation death axis is added by ferroptosis, which is regulated by the cystine/glutamate antiporter (system xC^−^)/reduced glutathione (GSH)/glutathione peroxidase 4 (GPX4) [55,56], and lipid peroxidation is a terminal biochemical event across multiple regulated cell death pathways [57]. The practical implication for ophiobolin medicinal chemistry is to profile caspases, vacuolisation or ER dilation, and ferroptosis markers across tumour states, e.g., epithelial–mesenchymal transition (EMT)-enriched breast cancer cells. This is because cell death pathway engagement varies with cellular context and can be altered by minor structural modifications [58]. Assessing OpA phenotypes benefits from the Nomenclature Committee on Cell Death (NCCD) system for classifying regulated cell death across studies, leading to caspase activation, plasma-membrane rupture, or lipid-ROS accumulation not being inferred from single markers [59]. Links between synthetic modifications and mechanisms are strengthened by multiparametric analyses such as these.

### 5.3. Modulation of Pro-Survival and Stress-Responsive Signalling Networks

OpA and medicinally altered derivatives regulate several important signalling axes instead of a single node in cancer-orientated in vitro studies. OpA was described to simultaneously inhibit phosphoinositide 3-kinase (PI3K)/mechanistic target of rapamycin (mTOR) and Ras/Raf/extracellular signal-regulated kinase (ERK) pathway output. This reduces the phosphorylation of protein kinase B (Akt), ribosomal protein S6 (S6) and ERK. Additionally, this diminishes cyclin-dependent kinases (CDKs) and retinoblastoma protein cell-cycle signalling, which are routinely used as phosphor markers in SAR work [60]. Alterations in Akt markers help position OpA analogues against pro-survival signalling pathways because PI3K-Akt generally supports proliferation and survival [61]. So that OpA-driven perturbations can activate both mitogenic and stress-activated pathways, mitogen-activated protein kinase (MAPK) modules, such as ERK, c-Jun N-terminal kinase (JNK), and p38, can combine growth cues with stress responses [62]. Sustained stress is often interpreted by tumour cells via the integrated stress response, which can steer adaptive signalling toward death when damage is excessive [63]. Marine-fungal metabolites often activate stress-response signalling rather than single linear pathways, supporting the interpretation of ophiobolins as multidimensional stress inducers [64]. A second regulatory mechanism is calcium homeostasis. OpA is a covalent CaM inhibitor; CaM-dependent signalling is associated with tumour migration and invasiveness [65]. CaM antagonism is also closely linked to cancer cell migration and stress adaptation, offering a mechanistic bridge between ophiobolin exposure and invasive phenotypes [66]. More precise analysis of Ca^2+^- and CaM-linked signalling effects is enabled by a less toxic OpA analogue developed as a CaM-directed chemical probe in K-Ras contexts [67]. Combining phospho-profiling with live Ca^2+^ flux assays strengthens mechanistic interpretation since altered Ca^2+^ signalling forms the basis of multiple key cancer features. Derivatives for combination therapy can therefore be selectively prioritised [68]. This can be done using combined phospho-signalling and Ca^2+^ flux profiling, in accordance with the integrated target and pathway structure summarised in Table 3.

## 6. In Vitro Biological Applications of Ophiobolin A and Related Derivatives

### 6.1. Anticancer Activity Across Tumour Models and Cellular States

OpA acts as a widely cytotoxic but mechanistically characteristic fungal sesterterpenoid. It shows low-micromolar to sub-micromolar inhibition of growth across glioma, melanoma, breast, and haematologic cell lines across in vitro panels. Heterogeneity is emphasised over a single dominant indication by multi-cell-line screening datasets and comparative phenotyping. In some cell lines clonogenicity is rapidly lost with widespread vacuolisation and suppressed migration, while others show stronger apoptosis- or autophagy-associated profiles. This activity is notable in glioblastoma models because it continues in apoptosis-resistant conditions, supporting derivative programmes and delivery concepts [13,20,39,46]. An important therapeutic consideration is represented by preferential activity against cancer stem-like cell populations. EMT, a programme linked to stemness, invasion, and resistance to several chemotherapies, increases cellular susceptibility to OpA-mediated killing in triple-negative breast cancer systems. It is suggested by flow cytometry and sphere-formation readouts that stem-associated subpopulations can be reduced by OpA. These subpopulations are identified by marker phenotypes, such as CD44 high or CD24 low. These findings are consistent with selective effects on plastic, tumour-initiating cell populations. Ongoing medicinal chemistry efforts support this objective; cancer stem cell (CSC)-directed activity is retained by simplified bicyclic derivatives and electrophile-adjusted derivatives, which also improve tractability and selectivity [23,25,38,58]. Cooperative effects with chemotherapeutic agents are less extensively characterised than OpA as a single compound, yet a clear, testable rationale exists. OpA-class compounds avoid apoptosis defects and stress-adaptation programmes that reduce the effects of many DNA-damaging drugs. They do this by promoting non-apoptotic death programmes and membrane or proteostasis stress. OpA, less toxic or acidity-responsive derivatives, should be paired with orthogonal stresses such as temozolomide, platinum drugs, or anthracyclines. In addition to this, dose–matrix synergy models should be used to quantify interaction. Following treatment withdrawal, CSC marker rebound should be tracked in systematic in vitro combination studies. When cooperative effects are observed, they are likely influenced by treatment scheduling. The threshold for DNA-damage lethality can be lowered by pre-stressing membranes or proteostasis, whilst recovery from OpA-induced organelle dysfunction may be inhibited by DNA damage. Formulations, such as OpA-loaded local delivery particles that can be coupled with systemic chemotherapy to reduce exposure, are also included in translation work. To avoid adding toxicity, any claimed synergy should be compared against controls [25,36,38,70]. Aside from OpA, natural ophiobolin congeners and semi-synthetic OpA derivatives support that the framework tolerates oxidation or state and stereochemical changes whilst largely maintaining low-micromolar strength, though individual ophiobolin congeners such as 6-epi-ophiobolin G have recently been evaluated in disease-specific cancer models, supporting therapeutic importance across the ophiobolin family [71]. New ophiobolin derivatives yielded from marine *Aspergillus* spp., such as 14,15-dehydro-6-epi-ophiobolin K, displayed sub-micromolar to low-micromolar GI_50_ (concentration causing 50% growth inhibition) values. These values varied across solid-tumour panels. This supported that peripheral functionalisation could maintain potent cytotoxicity while providing new sites for derivatisation [72]. Across broader congener series, submicromolar IC_50_ (half-maximal inhibitory concentration) values were reached by Ophiobolin T and 6-epi-ophiobolin G, emphasising that potency can be significantly affected by relatively small structural changes [73]. Cytotoxicity has also been reported for Ophiobolin K and 6-epi-ophiobolin K, suggesting that anticancer activity is retained within the ophiobolin family [74].

### 6.2. Anti-Proliferative and Anti-Metastatic Phenotypes

In addition to the direct induction of regulated cell death, OpA and structurally related derivatives display noticeable anti-proliferative effects. OpA achieves these effects by interfering with cell-cycle progression, and shows suppression of migratory and invasive behaviour in multiple cancer models [13,46,58]. For tumours in which metastatic spread instead of primary tumour burden alone regulates clinical outcome, these phenotypes are particularly relevant. Multiple in vitro studies report that OpA treatment induces cell-cycle arrest before evident cell death, suggesting that growth inhibition contributes alongside direct cell-killing activity. Exposure to OpA changes cell-cycle distribution in glioblastoma and melanoma cell lines, most often with G0/G1 or S-phase accumulation, controlled by cellular context and concentration. This shows that by interrupting coordinated cell-cycle progression, OpA can delay proliferation, resulting in restricted clonal expansion even in apoptosis-resistant populations [13,46]. For targeting slow-cycling treatment-tolerant cancer subpopulations, such growth restraint may be particularly valuable. Concurrently, anti-migratory and anti-invasive properties that are consistent with an anti-metastatic profile are displayed by OpA. Functional assays revealed that OpA treatment notably decreased wound closure and reduced both transwell migration and invasion through components of the extracellular matrix [13]. In glioblastoma and breast cancer models, where motility and invasiveness are closely associated with poor prognosis, these effects have been reported [13,58]. Notably, these phenotypes are observed at concentrations that do not instantly induce cell death; this supports the idea that OpA can detach migratory capacity from cell viability. Sensitivity to OpA varies across breast cancer cell populations. Better growth inhibition is shown by highly migratory and invasive cells after treatment compared to more proliferative, epithelial-like counterparts. This implies that OpA exhibits functional selectivity; rather than uniformly affecting bulk tumour viability, it preferentially suppresses cell states associated with invasion and metastatic potential [58]. These findings collectively position OpA as a molecular framework with dual anti-proliferative and anti-metastatic activity, justifying its continued development as a mechanistically novel lead for in vitro cancer research and derivative optimisation [35]. The anti-proliferative activity profile is expanded beyond the lead structure by a growing body of ophiobolin congeners. Drophiobolins A and B are examples of ophiobolin sesterterpenoids co-produced with OpA-related metabolites. They support the idea of selective structure edits that maintain growth suppression and bioactivity, while generating new SAR handles by adding oxidation-state and stereochemical diversity [75]. Similarly, additional templates for tuning the potency–toxicity balance were offered by Bipolaricins A-I, which introduce tetracyclic and spirocyclic motifs and were evaluated for cytotoxic endpoints [76]. In functional terms, due to calmodulin-related programmes regulating EMT, migration, and invasion, OpA-like derivatives that preserve target engagement can reasonably extend anti-metastatic phenotypes and be evaluated at sublethal doses in wound-healing and transwell assays [77].

### 6.3. Additional In Vitro Activities Relevant to Therapeutic Development

**Figure 5:**
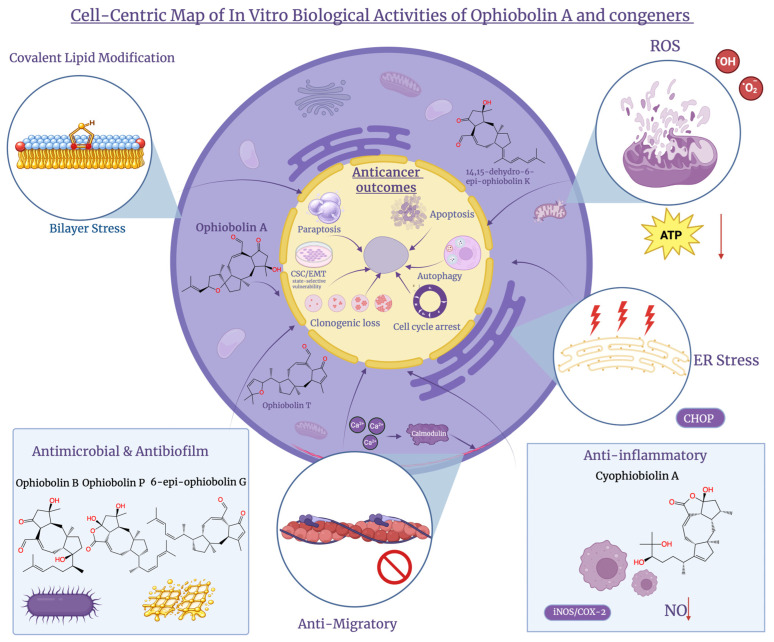
Cell-centric overview of in vitro activities of Ophiobolin A and congeners. Ophiobolins induce membrane lipid modification, ER stress, ROS and Ca^2+^/calmodulin disruption, converging on anticancer outcomes including apoptosis, paraptosis, autophagy, and clonogenic loss, alongside anti-migratory, antimicrobial, and anti-inflammatory effects. Created in BioRender. Wilk, I. (2026) https://BioRender.com/9v0pblc.

OpA and selected derivatives show several other in vitro activities that are significant when evaluating potential for therapeutic development. These activities also influence how chemists design analogues, for example, tuning the electrophilic aldehyde that drives covalent reactivity, beyond its well-known anticancer effects. Fungal growth is inhibited by several ophiobolin family members in vitro. For instance, Ophiobolin A and B demonstrate measurable activity against zygomycete fungi, with minimum inhibitory concentrations (MICs) reported in the low-to-mid µg/mL range depending on the species [78]. Selective antibacterial activity against *Enterococcus faecalis* has been shown by newer ophiobolin-type sesterterpenes, derived from *Bipolaris* [79]. Additionally, moderate antibacterial activity against Gram-positive pathogens such as methicillin-resistant *Staphylococcus aureus* (MRSA) has been displayed by congeners such as Ophiobolin P-T, whilst moderately strong activity against multiple Gram-positive strains and antibiofilm effects in vitro have been reported with 6-epi-ophiobolin G [79,80]. Anti-biofilm action is another distinct perspective, as shown in Figure 5. *Mycobacterium* biofilm formation can be inhibited by marine-derived ophiobolins, and isoniazid activity can be restored in vitro by suppressing biofilms instead of directly killing planktonic cells [81], as described in Table 4. Lipopolysaccharide (LPS)-induced inflammatory signalling is suppressed by several new ophiobolins in macrophage models. This significantly inhibits the production of nitric oxide (NO) and downregulates inducible nitric oxide synthase (iNOS)/cyclooxygenase-2 (COX-2) expression. These features are frequently used to prioritise semi-synthetic analogues with improved therapeutic windows [82]. NO-inhibitory and antibacterial effects are also shown by related metabolites, such as cyophiobolins, which suggests certain derivatives exhibit inflammation–infection dual relevance [83]. Neurologically, OpA is a covalent CaM inhibitor. This provides a mechanistic link to neuronal CaM-dependent signalling, by reacting with lysine residues and inactivating bovine brain CaM in vitro [84]. CaM-targeting ophiobolin-related electrophiles are occasionally discussed as chemical tools, because CaM regulates key brain pathways involved in excitability and plasticity, although wide reactivity and toxicity remain major limitations [85].

## 7. Preclinical Therapeutic Potential and Translational Challenges of Ophiobolin A

OpA and closely related ophiobolin structures are relevant in preclinical models because of their ability to kill tumour cells. They achieve this by mechanisms different from traditional DNA-damaging or microtubule-targeting anticancer drugs. OpA’s activity in chemo-resistant stem-like states is explained by being highlighted as a CSC target. OpA targets these cells through the disruption of K-Ras and calmodulin signalling, instead of targeting faster dividing cells first [86]. Ophiobolin chemistry enables more extensive covalent engagement of nucleophile-rich biomolecules, such as primary amines. This is a property that is increasingly used in probe and derivative design but also requires careful safety pharmacology [87]. Advantages of OpA over conventional cytotoxics include its potential efficacy in apoptosis-resistant contexts. Its potential for pathway-selective phenotypes, such as Ras- or CaM-linked stemness, instead of uniform mitotic arrest is also advantageous [88]. However, there are still preclinical limitations, such as selectivity and potency. Ophiobolins can have effects across a variety of eukaryotic cells. Therefore, therapeutic windows must be engineered by delivery, masking, or context-activated derivatives and benchmarked against normal-cell liabilities [89,90]. EMT-state-dependent redox responses reported for OpA imply that cell state may modify sensitivity. This is important for biomarker-led development [78,91].

**Figure 6:**
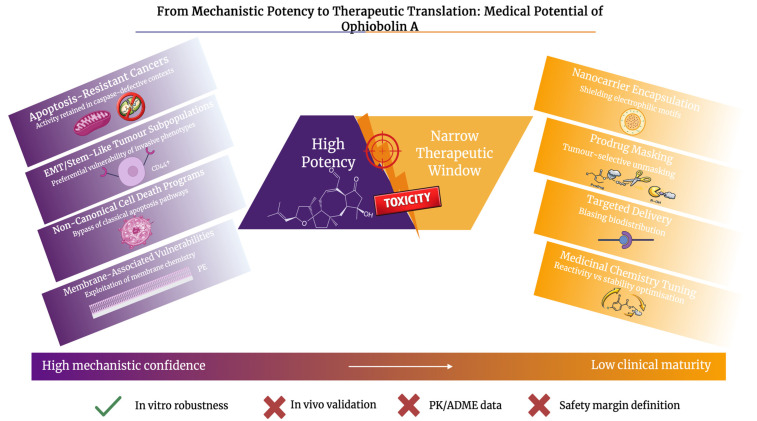
From mechanistic potency to therapeutic translation: medical potential of Ophiobolin A. This figure compares strong mechanistic and in vitro anticancer activity, including efficacy in apoptosis-resistant and invasive cancer phenotypes, with a tight therapeutic window led by toxicity. Translational strategies such as nanocarrier encapsulation, prodrug masking, targeted delivery, and medicinal chemistry optimisation are emphasised as routes to advance clinical feasibility. Created in BioRender. Wilk, I. (2026) https://BioRender.com/dfcedeo.

Toxicity, therapeutic window, stability, and delivery constraints dominate the translation of OpA and derivatives from potent in vitro cytotoxins into drug candidates. There is a risk of widespread, mechanism-independent reactivity in normal tissues implied by OpA’s electrophilic motifs. Recent experiences with covalent drugs show that to avoid off-target protein or lipid alkylation and dose-limiting toxicity, warhead reactivity must be tuned. Tuning can occur using kinetics, selectivity and exposure [92,93]. Therefore, when prioritising OpA derivatives, measuring and evaluating electrophile reactivity, including fragment or warhead profiling concepts, is essential [94]. Aldehyde or Michael chemistry can produce adducts with nucleophilic residues. Due to this, systemic inflammation or liabilities remain likely and careful in vivo tolerability studies are required [95]. The importance of defining exposure-response relationships and safety margins was emphasised in a small animal study. The conclusion of the study was that OpA can trigger rapid inflammatory mediator release [96]. Lipophilicity and electrophilic instability, such as thiol- or amine-rich matrices, are followed by stability and delivery limitations. These limitations motivate formulation-led approaches to regulate biodistribution and decrease peak systemic exposure [97]. This is shown in Figure 6. Pharmacokinetic control, which includes slower release and lower Cmax, is conceptually attractive for reactive natural products. This can be provided by regional delivery platforms such as drug-eluting beads or microspheres [98,99,100].

OpA is shown to covalently target mitochondrial respiratory complex IV, causing a metabolic collapse in cancer cells. This feature of OpA slows down clinical translation. While in metabolically stressed tumours this mechanism may be of benefit, complex IV is essential in high-energy normal tissues. As a result, concerns are raised over on-target mitochondrial toxicity and narrowing the therapeutic window. Therefore, delivery strategies that are exposure-controlled are crucial to balance antitumour activity with systemic tolerability. Additionally, early mitochondrial safety profiling will also be a key factor [12].

Nanocarrier strategies for OpA are a relatively recent development. The aim of nanocarrier strategies is to solubilise OpA and derivatives, protect reactive motifs until the drug reaches the tumour, and to shape biodistribution. Liposomes are commonly used as delivery platforms, as they have well-known clinical validation. Liposomes can increase circulation time, reduce free-drug peaks, and enable surface engineering. The most common liposome surface modifications are polyethylene glycol (PEG) modification (PEGylation) or ligand presentation for tumour-specific uptake [101]. When using hydrophobic terpenoids like OpA, stability and tumour accumulation can be enhanced using lipid/polymer nanoparticles or hybrid systems. This can be further supported by the literature about terpenoid nano-formulation [102]. A way to combine enhanced permeability and retention (EPR)-based accumulation with deeper tumour penetration is offered by size- or charge-switching designs. pH-responsive shrinkable nanoparticles [103] are a good example of this type of delivery system. Together, prodrug and targeted delivery can mask OpA’s most reactive handles. This can be done with cleavable promoieties that unmask especially in tumours. For example, promoieties can be unmasked within reactive oxygen species (ROS)-rich reductive or enzyme-high microenvironments. This reduces the risk of off-target reactivity [104,105]. This approach complements emerging OpA biology in cancer cells. Moreover, the therapeutic index can be widened by pairing it with ligand targeting [106,107]. This is summarised in Table 5.

## 8. Discussion

### 8.1. Mechanistic Integration of Ophiobolin A Cytotoxicity

Together, the evidence reviewed here supports the conclusion that OpA and its congeners are fungal-derived small molecules with distinct anticancer activity, able to participate in mechanisms past standard apoptosis. OpA was shown to form cytotoxic adducts by covalently reacting with membrane PE in unbiased genetic screens, causing disruption of lipid bilayers and contributing to the selective killing of cancer cells, supporting earlier work in human haploid cells [22]. Alongside membrane reactivity, targeting of mitochondrial complex IV subunits has been reported in recent chemoproteomic studies. The COX5A and HIGD2A subunits of complex IV are frequently targeted. This leads to oxidative stress, compromised membrane potential, and ATP reduction [12]. The wider recognition that disrupting redox balance and membrane integrity can overthrow apoptotic resistance is supported by understandings of these core mechanisms, and they highlight the value of OpA as a molecular probe for alternative death pathways.

### 8.2. Ophiobolin A and Non-Apoptotic Cell Death Pathways

Many of the cellular responses caused by OpA align with established non-apoptotic cell death programmes. For instance, ferroptosis is characterised by iron-dependent lipid peroxidation and lethal membrane rupture; this is a mode of death separate from caspase-dependent apoptosis and progressively pursued for therapeutic gain in oncology [108,109]. Though direct evidence linking OpA cytotoxicity to canonical ferroptosis is yet to be established, the significance of membrane lipid interactions, mitochondrial dysfunction, and redox perturbation situates OpA within the conceptual framework of therapies that leverage ferroptotic vulnerabilities [108]. Canonical ferroptosis validation approaches should be applied to studies in the future to address this. These approaches include rescue experiments using ferroptosis inhibitors, like ferrostatin-1 or liproxstatin-1 [110], the assessment of iron dependency, and the quantification of lipid peroxidation using boron-dipyrromethene (BODIPY)-C11 oxidation. In addition, observing the activity of GPX4, glutathione decrease, and the function of system xC^−^ [111] would help to establish if OpA-induced cytotoxicity engages ferroptotic pathways.

### 8.3. Limitations and Knowledge Gaps

**Figure 7:**
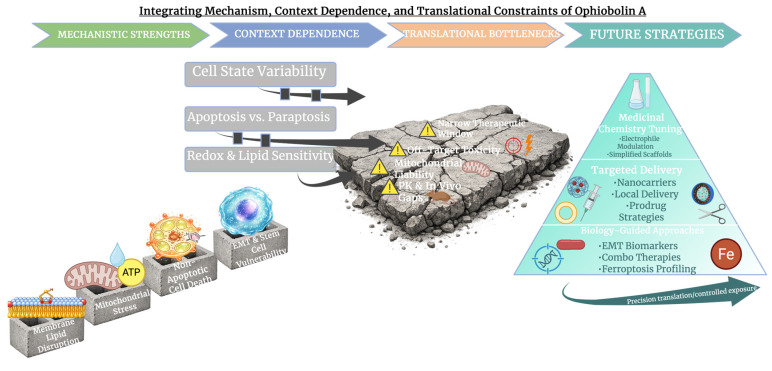
Mechanistic strengths, context dependence, and translational challenges of Ophiobolin A. Membrane- and mitochondria-based mechanisms support non-apoptotic cancer cell killing but continue to be highly context-dependent, collectively revealing toxicity and pharmacokinetic limitations that encourage targeted, chemistry- and biology-guided translational strategies. Created in BioRender. Wilk, I. (2026) https://BioRender.com/ao6hz9a.

Significant limitations temper interpretation and translational promise, despite this convincing mechanistic landscape. Unfavourably, almost all available data is derived from in vitro models, restricting the confidence in the physiological relevance of the observed mechanisms, as depicted in Figure 7. Although one early study reported the antitumour effects of OpA in a mouse melanoma lung metastasis model, the data remains partial and has not been greatly replicated or supported through pharmacological characterisation [7]. Moreover, comprehensive pharmacokinetic (PK) and pharmacodynamic profiling of OpA and congeners is still absent, leaving many questions about bioavailability, metabolic stability, tissue distribution, and therapeutic index unanswered. Crucially, the OpA core seems to have a narrow therapeutic window. This is most likely a result of its intrinsic electrophilic character, which may push for nonspecific reactivity with biomolecules within cells. Its electrophilic character may also strongly contribute to its off-target toxicity. The highly reactive 1,4-dicarbonyl motif is able to form covalent adducts with nucleophilic residues, like primary amines and thiols. This has the potential to disrupt membrane lipids and proteins past deliberate targets. Basic cellular functions may be impaired by this limited selectivity, and the prediction of safety margins may become complicated. These disadvantages are still poorly charcterised in vivo, which limits current evaluation of safety, selectivity and clinical viability. Assessing if strong in vitro effects can change into effective in vivo exposures without this information is hypothetical at best.

### 8.4. Future Directions for Medicinal and Translational Development

The OpA scaffold presents opportunities for medicinal chemistry optimisation from a drug development perspective. For example, semi-synthetic alterations led by pharmacophore-directed retrosynthesis have produced simplified bicyclic derivatives with preserved anticancer activity, demonstrating that structural complexity and electrophilic warheads can be adjusted to control effectiveness and selectivity [38]. SAR studies should be integrated with rigorous absorption, distribution, metabolism, and excretion (ADME) characterisation in future work to identify derivatives with improved safety and pharmacological properties. Emerging research directions expand the relevance of OpA beyond classical cytotoxicity. Systemic evaluation of lipid peroxidation data, iron dependency, and interaction with redox signalling systems would help to explain how OpA and its analogues activate these mechanisms and whether they can be used advantageously in precision oncology, given the growing attention around ferroptosis and other non-apoptotic death pathways as therapeutic targets [108]. In summary, OpA displays interesting mechanistic delivery and anticancer activity in cell-based models. However, addressing current gaps in in vivo validation, pharmacokinetic interpretation, and focused medicinal chemistry optimisation will be necessary to advance OpA toward therapeutic application.

## Figures and Tables

**Table 1:** Key structural elements of Ophiobolin A: biosynthetic origin, chemical reactivity, and functional consequences. Abbreviations: flavin adenine dinucleotide (FAD), mechanism of action (MoA).

Structural Element or Motif (OpA)	Likely Biosynthetic Origin	Evidence and Examples	Chemical Reactivity Readout	Functional/Biological Consequence
C25 sesterterpene backbone (GFPP-derived) [8]	Bifunctional sesterterpene synthase, prenyltransferase and terpene cyclase domains, forms GFPP and triggers cyclisation cascade	Multi-cluster contribution to ophiobolin biosynthesis described in *A. ustus*	Determines scaffold for downstream oxidation and electrophile placement	Enables dense, rigid 3D framework supporting potent phenotypes
Signature 5–8–5 tricyclic core [28]	Cyclisation or rearrangement cascade catalysed by ophiobolin/sesterterpene synthase	Genomic or biochemical evidence that core assembly is synthase-led and reviewed gene-cluster logic	Conformational constraint and positions electrophiles	Privileged topology for bioactivity and crucial for SAR retention
Electrophilic enal–ketone, “1,4-dicarbonyl-like” hotspot [28]	Installed or tuned by tailoring oxidations, e.g., P450s or FAD-dependent oxidases, that alter oxidation state and electrophilicity	Reviews of ophiobolin clusters emphasise synthase and oxidoreductases as core toolkit	Reacts with primary amines and forms pyrrole-type adduct chemistry	Key determinant of potency and selectivity in many OpA-focused MoA or SAR discussions
Covalent adduct formation with phosphatidylethanolamine (PE) [22]	Not a biosynthetic step, but a downstream chemical fate facilitated by the electrophilic motif	In human cells, OpA forms pyrrole-containing PE adducts, lowering PE biosynthesis mitigates toxicity	Direct covalent lipid adduct formation (membrane destabilisation)	Mechanistic explanation for cytotoxicity linked to membrane integrity
Synthetic access, benchmark syntheses supporting stereodefined probes and derivatives [30,31]	Chemical (non-biosynthetic): total synthesis routes validate structural assignments and enable analogue design	Enantioselective total synthesis of (+) -OpA and concise 14-step synthesis of (+) -6-epi-OpA	Enables systematic SAR and probe installation	Supports medicinal chemistry and mechanistic tool compounds

**Table 2:** Synthetic and semi-synthetic functionalisation map for OpA, describing what has been altered, how, and which biological or chemical readouts support these design choices. Abbreviations: calmodulin (CaM), C/EBP homologous protein (CHOP), glioblastoma (GBM).

Chemistry Lever or Derivative Class	Structural Change or Handle	Typical Methods Used	Design Intent	Key Readouts Reported
Benchmark: full scaffold access [30]	Enantioselective access to (+) -OpA framework	Convergent total synthesis featuring spiro-ring construction and medium-ring closure	Enable stereodefined supply, probe design, and systematic SAR that is difficult from isolation alone	First fully controlled synthetic access to (+) -OpA, facilitates analogue programmes
Benchmark: epi-series access [31]	(+) -6-epi-OpA (stereochemical perturbation at C6)	Concise total synthesis	Provide controlled access to epi-variants to test stereochemical sensitivity of activity	Demonstrates feasibility of quick access to epi-framework used in SAR comparisons
Electrophile-to-function linkage [22]	Native OpA electrophile set engages phosphatidylethanolamine (PE)	Cellular lipid adduct detection and mechanistic profiling	Prove if OpA cytotoxicity can arise from covalent lipid modification	Formation of pyrrole-containing PE–OpA adducts, PE depletion mitigates cytotoxicity
Paraptosis trigger [13]	Native OpA (no derivatisation required)	Cell biology in GBM models	Determine a clinically relevant phenotype: non-apoptotic death in apoptosis-resistant contexts	Paraptosis-like vacuolisation or cytoskeletal effects in glioblastoma models
ER stress pathway refinement [39]	Native OpA, pathway dissection	Mechanistic assays, ER stress markers and inhibitor blocks	Clarify which stress axes are needed for paraptosis-like death	CHOP-mediated ER stress involvement, thiol antioxidants can block effects
Classic target class: calmodulin inhibition [9]	OpA vs. fewer active analogues, e.g., anhydro forms	Biochemical enzyme assay, CaM-activated PDE	Link electrophile or structure to a defined protein pathway historically tied to OpA	Potent inhibition of calmodulin-activated PDE, less active analogues inhibit less
Covalent protein engagement mapping [11]	Specific CaM lysines bind OpA	Mutagenesis and binding or inhibition characterisation	Demonstrate residue-level covalent binding logic, supporting electrophile hotspot concept	Lys-75, Lys-77, Lys-148 implicated in binding stoichiometry
In vivo + “unique chemistry” framing [36]	OpA plus natural or modified comparisons, including pyrrolylation chemistry	In vivo evaluation and SAR discussion	Bridge potent in vitro to in vivo plausibility and highlight primary-amine reactivity	NCI-60 potency context, emphasises unusual pyrrolylation or amine reactivity as mechanistic clue
Electrophile tuning at aldehyde or ketone [23]	Systematic variation of ketone and unsaturated aldehyde	Semi-synthesis, selective redox or derivatisation	Alter stability or handling while mapping how much electrophilicity is required for activity	Cytotoxicity shifts in breast and glioblastoma stem models when electrophile is altered
Microenvironment targeting [25]	Acid-sensitive OpA analogues	Semi-synthesis of acid-labile or acid-tuned derivatives	Improve selectivity by exploiting tumour-relevant acidity (GBM microenvironment)	Enhanced activity under tumour-relevant acidic conditions vs. neutral conditions
Multimeric covalent “crosslinkers” [35]	Dimeric or trimeric unsaturated ester OpA analogues	Linker-based synthesis (multimerisation)	Attempt to crosslink primary-amine targets while preserving pyrrolylation capability	Retained pyrrolylation ability, single-digit µM potencies in panels of cancer cell lines
Cell-line-dependent death modality [40]	Native OpA across diverse mammalian cancer origins	Comparative cell death phenotyping	Note that MoA may not be singular, supports need for derivative probes	Distinct death mechanisms depending on cancer cell origin
Plant pathogen congener context (activity shifts in anhydro/epi forms) [41]	Natural anhydro or hydroxy variants (structure perturbations)	Isolation and bioassays	Provide natural SAR hints that guide which positions tolerate change	Bioactivity present but often altered, supports stereochem or oxidation sensitivity

**Table 3:** Molecular targets and mechanistic nodes engaged by OpA in mammalian cells: direct covalent interactions, pathway outputs, and phenotypic consequences. Abbreviations: large-conductance Ca^2+^-activated potassium channel (BKCa), cancer stem cell (CSC), liquid chromatography–tandem mass spectrometry (LC-MS/MS), oxygen consumption rate (OCR), retinoblastoma protein (RB), cytochrome c oxidase subunit 5A (COX5A), hypoxia-inducible gene domain family member 2A (HIGD2A), mitochondrial membrane potential (ΔΨm).

Molecular Target or Node	Engagement Type (Direct or Indirect) and Covalent Site If Known	Key Experimental Readouts	Main Phenotype and Interpretation
Phosphatidylethanolamine (PE) [22]	Direct or covalent: OpA reacts with ethanolamine headgroup, forming pyrrole-containing PE–OpA adducts	Lipid adduct detection (LC–MS/MS), PE depletion or genetics rescue, membrane destabilisation metrics	Membrane stress or bilayer destabilisation as a proximal driver of cytotoxicity
Calmodulin (CaM) [11,69]	Direct or covalent: Lys adduction, notably Lys-75 as primary inhibition site, and Lys-77 or Lys-148 binding contributions	Site-directed mutagenesis, CaM-binding stoichiometry, CaM-modulated enzyme inhibition kinetics	CaM inactivation leads to a plausible route to cytoskeletal or signalling disruption
BKCa (large-conductance Ca^2+^-activated K^+^ channel) [13]	Reported as a functional node in GBM paraptosis context, mechanistic connection via Ca^2+^ and CaM and ion homeostasis is discussed	BKCa activity assays or electrophysiology, vacuolisation scoring, caspase independence	Paraptosis-like death in glioblastoma, vacuolisation and cytoskeletal remodelling
ER proteostasis or thiol reactivity → ER stress (CHOP) [39]	Indirect or proximal chemistry: covalent modification of protein thiols suggested, leading to misfolded proteins and ER stress	ER dilation imaging, CHOP or ER stress markers, rescue with thiol antioxidants	ER-derived vacuolisation and paraptosis-like death connected to proteostasis stress
PI3K/mTOR, Ras/Raf/ERK, CDK/RB signalling outputs [60]	Indirect: frequently shown via reduced phosphorylation of pathway effectors	Phospho-S6, phospho-ERK, phospho-RB and apoptosis or cell-cycle assays	Multi-pathway suppression consistent with broad anticancer phenotypes
EMT or stem-like state dependence [58]	Context-dependent: cell state alters sensitivity, not a target, but critical for MoA framing	EMT marker state, cytotoxicity shifts, CSC-like markers, e.g., CD44 or CD24	Higher susceptibility in EMT-enriched breast cancer models, useful for positioning or selectivity
Mitochondrial respiratory complex IV (COX) [12]	Direct or proximal: covalent engagement of complex IV subunits, e.g., COX5A and HIGD2A reported	Chemoproteomics, OCR and ATP depletion, ΔΨm loss, ROS accumulation	Bioenergetic collapse and redox stress that potentiate apoptosis-independent cell death

**Table 4:** Candidate clinical applications of OpA and ophiobolin-family derivatives. Abbreviations: antibody–drug conjugate (ADC), pharmacokinetics (PK).

Candidate Clinical Application	Compound(s) to Compare	Natural Source or Producing Fungi	Structure “Handle” That Differentiates from OpA	Most Supported Mechanism Node	In Vitro Evidence from Section 5	Translation Lever	Development Status and Key Limitations
**Glioblastoma (GBM), apoptosis-resistant disease [13,22,36]**	OpA	Plant-associated fungi, incl. *Bipolaris* spp.	Benchmark 5–8–5 core and electrophilic enal or ketone, 1,4-dicarbonyl-like motif	Paraptosis-like death, ER stress, also membrane lipid engagement (PE adducts)	Vacuolisation or paraptosis markers, caspase independence, migration suppression	Local delivery, reduce systemic exposure or tumour-state targeting (microenvironment)	Preclinical only, liabilities: electrophilicity and off-targets, poor solubility
**GBM, microenvironment-selective killing [25]**	Acid-tuned OpA analogues (semi-synthetic)	Semi-synthetic (OpA starting material)	Acid-responsive or acidity-enhanced activity while preserving reactive pharmacophore	Same OpA death biology, but selectivity biased by pH	Cytotoxicity shifts at tumour-relevant pH, GBM-focused panels	Smarter selectivity without sacrificing potency, pairing with GBM delivery strategies	Preclinical, still electrophile-driven, needs PK and tox window
**Breast cancer with EMT or CSC-like state [38,58]**	OpA and simplified bicyclic OpA derivatives	OpA: fungal origin, simplified derivatives: synthetic and semi-synthetic	Pharmacophore-guided simplification, retain killing, enhance tractability	EMT state increases susceptibility, membrane or proteostasis stress axes suggested	Sphere formation, CSC markers, CD44 high or CD24 low, EMT state stratification	Patient stratification by EMT or stemness marker, combine with standard chemo	Preclinical, must prove therapeutic index and delivery feasibility
**Rhabdomyosarcoma or solid tumours amenable to loco-regional therapy [70]**	OpA-loaded chemoembolisation particles	Formulation-based (OpA payload)	Not a structure change, delivery platform is the differentiator	Cytotoxic payload effect, goal is tumour-local exposure	Cell viability reduction in RD line, formulation release characterisation	Interventional radiology-style local delivery to widen safety margin	Preclinical formulation study, needs in vivo efficacy/tox and scale-up
**Hematologic malignancies [47]**	Ophiobolin A, B, C and K	Multiple fungi including *Aspergillus* spp. and plant pathogens, e.g., *Bipolaris* and *Drechslera*	Oxidation pattern and stereochemical variation, sometimes epi- or anhydro series	Often apoptosis, dominant phenotypes in leukaemia models	Low IC50 growth inhibition, apoptosis markers	Candidate for payload use, ADC or linker concepts, rather than systemic small molecule	Preclinical, selectivity and tox major restriction
**Chemo-resistant breast cancer [48,49,50]**	Ophiobolin O	*Aspergillus ustus*-associated isolates in the literature	Characteristic oxidation or unsaturation pattern vs. OpA (family divergence)	MAPK-linked apoptosis and cell-cycle arrest, resistance reversal reported	Cell-cycle distribution, apoptosis, sensitisation readouts	Combination therapy logic (schedule-dependent)	Preclinical, mechanism differs from OpA, still lacks tox profiling
**Anti-biofilm adjunct (tuberculosis and *Mycobacterium*) [80,81]**	Marine-derived ophiobolins and 6-epi-ophiobolin G	Marine-derived fungi, reported as marine-derived ophiobolins	Congener-dependent, goal is phenotype (biofilm) not cytotoxicity	Biofilm inhibition (adjunctive antimicrobial strategy)	Biofilm assays, potentiation of antibiotics	Adjunct concept, lower dose, synergy	Early preclinical, specificity and host–cell tox need separation
**Anti-inflammatory lead lane (macrophage NO, iNOS, COX-2 readouts) [82]**	New ophiobolins from deep-sea fungi	*Aspergillus* spp. deep-sea-derived	New oxidation patterns, often screened for NO inhibition	Downregulation of inflammatory mediators, NO/iNOS and COX-2	LPS-stimulated macrophage assays	Could be a non-oncology application if cytotoxicity can be decoupled	Early discovery, needs selectivity vs. cytotoxicity

**Table 5:** Biological constraints and translational rationales governing therapeutic exploration of OpA and derivatives.

Biological or Pathological Constraint	Why Standard Therapies Fail in This Context	Ophiobolin Property That Becomes Advantageous	Dominant Liability Introduced by Ophiobolins	Risk-Mitigation or Translational Strategy	Representative Evidence Base
Apoptosis-refractory tumour states [39]	Caspase inactivation or apoptotic pathway rewiring limits efficacy of DNA-damaging or mitotic agents	Ability to induce non-apoptotic death via membrane stress, ER stress, and paraptosis-like pathways	Widespread cytotoxicity due to electrophilic reactivity	Local or regional delivery, dose-sparing formulations	Paraptosis-like death and PE adduction studies in GBM models
EMT-high/stem-like cellular states [58]	Plastic, slow-cycling populations avoid cytotoxic and targeted therapies	Sensitivity of EMT or CSC states to redox, membrane, and proteostasis stress	Poor selectivity between malignant and normal plastic cells	Biomarker-guided patient stratification, combination scheduling	EMT-dependent sensitivity and CSC depletion studies
Multi-pathway redundant survival signalling [60]	Parallel PI3K-, MAPK-, and Ca^2+^-dependent survival pathways only compensate when a single node is inhibited	Concurrent disruption of CaM signalling, lipid homeostasis, and stress-response pathways can reduce pathway repitition	Mechanistic promiscuity complicates target assignment and saftey profiling	Context-restricted use, supported by mechanistic deconvolution with less reactive probes	Phospho-signalling, CaM inhibition, and stress-response literature
Biofilm-protected microbial communities [81]	Biofilm architecture confers tolerance rather than classical resistance	Membrane-active chemistry disrupts biofilm integrity without relying on growth inhibition	Host–cell toxicity at bactericidal concentrations	Adjunctive, low-dose use with antibiotics	Biofilm suppression and antibiotic-potentiation studies
Hyper-inflammatory macrophage activation [82]	LPS-driven NO-, iNOS-, COX-2-, and cytokine-linked signalling limits the efficacy of closely pathway-specific inhibitors	Ability to suppress iNOS and COX-2 expression and reduce NO production through wider stress modulation	Anti-inflammatory activity may overlap with general cytotoxicity, narrowing the therapeutic window	Lead optimisation and dose-window profiling to seperate electrophilicity from anti-inflammatory signalling effects	Macrophage NO-inhibition and iNOS/COX-2 suppression studies
High-energy-demand tissues and metabolically active tumours [12]	Dependence on intact mitochondrial respiration restricts therapeutic selectivity, as both tumour and normal high-energy tissues are exposed to respiratory distruption	Metabolic collapse in susceptible tumour cells can be trigged by covalent engagment of mitochnodrial respiratory machinery	Risk of on-target mitochondrial toxicity in normal tissues	Exposure-controlled delivery and early mitochondrial safety profiling	Chemoproteomic evidence for mitochnodrial complex IV engagment and related toxicity concerns

## Data Availability

No new data were created.

## Abbreviations

The following abbreviations are used in this manuscript:

ADME	Absorption, distribution, metabolism, and excretion
ADC	Antibody–drug conjugate
Akt	Protein kinase B
AIP-1/Alix	Apoptosis-inducing protein 1
ATP	Adenosine triphosphate
BGC	Biosynthetic gene cluster
BKCa	Large-conductance Ca^2+^-activated potassium channel
Ca^2+^	Calcium ion
CaM	Calmodulin
CDK	Cyclin-dependent kinase
CHOP	C/EBP homologous protein
COX	Cytochrome c oxidase (mitochondrial complex IV)
COX-2	Cyclooxygenase-2
COX5A	Cytochrome c oxidase subunit 5A
CSC	Cancer stem cell
ΔΨm	Mitochondrial membrane potential
EMT	Epithelial–mesenchymal transition
ER	Endoplasmic reticulum
ERK	Extracellular signal-regulated kinase
EPR	Enhanced permeability and retention
FAD	Flavin adenine dinucleotide
GBM	Glioblastoma
GFPP	Geranylfarnesyl diphosphate
GI50	Concentration causing 50% growth inhibition
GPX4	Glutathione peroxidase 4
GSH	Reduced glutathione
HIGD2A	Hypoxia-inducible gene domain family member 2A
iNOS	Inducible nitric oxide synthase
IC50	Half-maximal inhibitory concentration
JNK	c-Jun N-terminal kinase
LC–MS/MS	Liquid chromatography–tandem mass spectrometry
LPS	Lipopolysaccharide
MAPK	Mitogen-activated protein kinase
MIC	Minimum inhibitory concentration
mTOR	Mechanistic target of rapamycin
MRSA	Methicillin-resistant *Staphylococcus aureus*
MoA	Mechanism of action
NCCD	Nomenclature Committee on Cell Death
NO	Nitric oxide
OpA	Ophiobolin A
OCR	Oxygen consumption rate
P450	Cytochrome P450 monooxygenase
PE	Phosphatidylethanolamine
PI3K	Phosphoinositide 3-kinase
PK	Pharmacokinetics
PMC	PubMed Central
RB	Retinoblastoma protein
ROS	Reactive oxygen species
SAR	Structure–activity relationship
S6	Ribosomal protein S6
